# Supplementary material for: Investigating student collaborative problem-solving competency and science achievement with multilevel modeling: Findings from PISA 2015
Source: PLoS One. 2023 Dec 8;18(12):e0295611. doi: 10.1371/journal.pone.0295611 (PMC10707649; doi:10.1371/journal.pone.0295611)
Supplement: S1 File — (DOCX) [file pone.0295611.s001.docx]

**S1 File. Measures Selected from PISA2015 Student Questionnaire for the Present Study**

**Collaboration Dispositions**

To what extent do you disagree or agree with the following statements about yourself? (“Strongly agree”, “Agree”, “Disagree”, “Strongly disagree”)

**(a) Valuing Relationships**

1. I am a good listener.
2. I enjoy seeing my classmates be successful.
3. I take into account what others are interested in.
4. I enjoy considering different perspectives.

**(b) Valuing Teamwork**

1. I prefer working as part of a team to working alone.
2. I find that teams make better decisions than individuals.
3. I find that teamwork raises my own efficiency.
4. I enjoy co-operating with peers.

**Inquiry-based Science Instruction**

When learning <school science> topics at school, how often do the following activities occur? (“In all lessons”, “In most lessons”, “In some lessons”, “Never or hardly ever”)

1. Students are given opportunities to explain their ideas.
2. Students spend time in the laboratory doing practical experiments.
3. Students are required to argue about science questions.
4. Students are asked to draw conclusions from an experiment they have conducted.
5. The teacher explains how a science idea can be applied to a number of different phenomena (e.g. the movement of objects, substances with similar properties).
6. Students are allowed to design their own experiments.
7. There is a class debate about investigations.
8. The teacher clearly explains the relevance of science concepts to our lives.
9. Students are asked to do an investigation to test ideas.

**Science Self-efficacy**

How easy do you think it would be for you to perform the following tasks on your own? (“I could do this easily”, “I could do this with a bit of effort”, “I would struggle to do this on my own”, “I couldn’t do this”.)

1. Recognise the science question that underlies a newspaper report on a health issue.
2. Explain why earthquakes occur more frequently in some areas than in others.
3. Describe the role of antibiotics in the treatment of disease.
4. Identify the science question associated with the disposal of garbage.
5. Predict how changes to an environment will affect the survival of certain species.
6. Interpret the scientific information provided on the labelling of food items.
7. Discuss how new evidence can lead you to change your understanding about the possibility of life on Mars.
8. Identify the better of two explanations for the formation of acid rain.

**Enjoyment of Science**

How much do you disagree or agree with the statements about yourself below? (“Strongly agree”, “Agree”, “Disagree”, “Strongly disagree”)

1. I generally have fun when I am learning <broad science> topics.
2. I like reading about <broad science>.
3. I am happy working on <broad science> topics.
4. I enjoy acquiring new knowledge in <broad science>.
5. I am interested in learning about <broad science>.

**Epistemological Beliefs**

How much do you disagree or agree with the statements below? (“Strongly agree”, “Agree”, “Disagree”, “Strongly disagree”)

1. A good way to know if something is true is to do an experiment.
2. Ideas in <broad science> sometimes change.
3. Good answers are based on evidence from many different experiments.
4. It is good to try experiments more than once to make sure of your findings.
5. Sometimes <broad science> scientists change their minds about what is true in science.
6. The ideas in <broad science> science books sometimes change.

**Interest in Broad Science Topics**

To what extent are you interested in the following <broad science> topics? (“Not interested”, “Hardly interested”, “Interested”, “Highly interested”, “I don’t know what this is”)

1. Biosphere (e.g., ecosystem services, sustainability).
2. Motion and forces (e.g., velocity, friction, magnetic and gravitational forces).
3. Energy and its transformation (e.g., conservation, chemical reactions).
4. The Universe and its history.
5. How science can help us prevent disease.

**Index of Economic, Social, and Cultural Status (ESCS)**

The ESCS was a composite score derived from three indicators, including home possessions, highest parental occupational status, and highest parental educational level.

Home possessions was a summary index of 25 household and possession items, with higher scores indicating more home possessions.

Highest parental occupational status was determined by analyzing responses to open-ended questions, which were subsequently coded into four-digit ISCO codes and mapped to the international socio-economic index of occupational status.

Highest parental educational level was determined by standardizing the parents’ highest education level based on four categories and converting it into estimated years of schooling.
