# Supplementary material for: Investigating student collaborative problem-solving competency and science achievement with multilevel modeling: Findings from PISA 2015
Source: PLoS One. 2023 Dec 8;18(12):e0295611. doi: 10.1371/journal.pone.0295611 (PMC10707649; doi:10.1371/journal.pone.0295611)
Supplement: Attachment — Submitted filename: Response to Reviewers.docx [file pone.0295611.s002.docx]

October 14, 2023

Dear Dr. Sonia Vasconcelos,

Thank you for your letter. The comments and suggestions provided by the editor and reviewers have significantly strengthened our paper. We greatly appreciate the editors’ and reviewers’ efforts. In response to the feedback we received, we have carefully addressed each comment below and made the necessary changes to the original manuscript. Any revisions resulting from these comments are clearly shown in blue font in the manuscript. Additionally, we changed APA format to Vancouver format and revised all the citations and the “References” section. Given that this is just a format change, we did not use blue highlight.

Reviewer #1:

The present study evaluates the contribution of collaborative problem-solving (CPS) competency to secondary education. By analyzing the Programme for International Student Assessment (PISA 2015), the authors examined factors that influence CPS competency and the relation of inquiry-based science instruction (IBSI) with science achievement. Data from 52 countries were included in the study providing a global view on the factors that matters for CPS and science achievements. Interestingly the data showed that CPS presented positive correlation with science achievements pointing out to the importance of integration CPS into teaching practices. Regarding IBSI, different activities performed by the teachers have positive and negative correlation with science performance among the students.

The studies present limitations, and some of them are clearly presented in the discussion section by the authors. But, as stated, as a starting point to address this important topic, the present study is a very good beginning.

Responses: We appreciate your thoughtful evaluation of our work and your positive feedback. We have made some changes to improve the quality of our manuscript as per your suggestions.

Minor points:

-Author should include schemes in the discussion section to summarize their results, such as what practices/activities correlates positive/negative with CPS and IBSI.

Responses: Thanks for the reviewer’s comment. We provided a summary of our results and added it to the first paragraph in the “Discussion and conclusions” section (Page 21, lines 15-18).

-Is it possible to have an overview on each country data and how they correlate with CPS/IBSI and science achievements? Is there any outlier in the analysis?

Response: We agree with the reviewer that including country-specific results would provide more details to the readers; however, given that 52 countries were included in our study, presenting results for each country in this manuscript could be overwhelming to readers.

Regarding outliers, we conducted outlier checks for each of the outcome variables by selecting 3 plausible values (PVs) out of the available 10 PVs and using histograms, z-scores, and Mahalanobis Distance as diagnostic tools. We did not include the outlier information in our manuscript because outliers did not affect our results.

In summary, the histograms (please see attached below) indicated that the outcome variables exhibited approximately normal distributions. The z-scores and Mahalanobis Distance measures showed some potential outliers. We conducted analyses with and without outliers, and the conclusions remained consistent in both scenarios. So we decided to use the full sample size, which would be more representative.

-It is sometimes hard to follow how raw data were converted into the data presented in the models. Mainly to the ones who do not know about statistics but have interest in this subject and in science education.

Response: We did not provide a detailed description of how raw data was converted, because data transformation was conducted by PISA researchers and the process has been documented in detail within the PISA reports. However, we have incorporated additional sentences in the Methods section (Page 10, lines 6-8, lines 11-14; Page 11, lines 10-11; Page 13, lines 8-10) and the Supporting Information file S1 (Page 2) to enhance clarity for readers.

-Do the authors think that CPS could have impact in other disciplines?

Response: That is a great question. We have now added two sentences regarding the potential impacts of CPS in other disciplines (Page 24, lines 1-4).

-It is not very clear why there are differences between valuing relationships and valuing teamwork to outcomes of the present investigation. They seem to correlate to each other.

Response: Thanks for the reviewer’s comment. We have now incorporated the correlation between valuing relationships and valuing teamwork into the manuscript. These two dimensions have a moderate correlation (*r* = 0.45), suggesting that they are different in terms of their conceptualization (Page 22, lines 8-12). While we did provide some initial explanations for this finding (Page 22, lines 11-21), we acknowledge that more in-depth research is needed to delve further into these relationships.

Reviewer #2:

The authors have done a fully revision of the manuscript. The authors have attended to the concerns highlighted by the reviewers. Although the authors have not provided the contents of the REU program, they addressed the Vygotsky's social constructivism theory.

The study was done with PISA 2015 data. Using the multilevel data the study aimed to investigate the relationship between collaboration dispositions and students’ collaborative problem-solving (CPS) competency. Also, CPS competency and inquiry-based science instruction were related to science achievement. Some novel data were highlighted by controlling some of the multilevel factors. For example, the study unveiled an important data that was masked by the HDI (countries indexes) effect, so that for some countries female students scores are higher than male students scores, showing that female students have more CPS competency than males.

Responses: Thank you for taking the time to review our manuscript and offering valuable feedback. The comments you provided before have been immensely helpful in improving our manuscript, and we appreciate your continued review of our work.
